# Impact of whole-body computed tomography on mortality and surgical management of severe blunt trauma

**DOI:** 10.1186/cc11375

**Published:** 2012-06-11

**Authors:** Jean-Michel Yeguiayan, Anabelle Yap, Marc Freysz, Delphine Garrigue, Claude Jacquot, Claude Martin, Christine Binquet, Bruno Riou, Claire Bonithon-Kopp

**Affiliations:** 1 Université de Bourgogne, Faculté de médecine, 21079 Dijon Cedex, France; 2 Centre Hospitalier Universitaire de Dijon, Département de Médecine d'Urgence, 7 Bd Jeanne d'Arc, BP77908 21079 Dijon Cedex, France; 3 Fédération des Urgences - SAMU 59, Centre Hospitalier Régional Universitaire de Lille, Avenue Oscar Lambert, 59037 Lille Cedex, France; 4 Pôle Anesthésie Réanimation, CHU de Grenoble, 38043 La Tronche cedex, France; 5 Université de la Méditerranée, Centre de traumatologie et Département d'Anesthésie Réanimation, Centre Hospitalier Universitaire Nord, Boulevard Pierre Dramard, 13015 Marseille, France; 6 INSERM CIE 01, Centre d'Investigation clinique-Epidémiologique clinique du CHU de Dijon, 7 Bd Jeanne d'Arc, 21079 Dijon Cedex, France; 7 Université Pierre et Marie Curie-Paris 6, Service d'Accueil des Urgences, GH Pitié-Salpêtrière, Assistance Publique-Hôpitaux de Paris, 75013 Paris, France

## Abstract

**Introduction:**

The mortality benefit of whole-body computed tomography (CT) in early trauma management remains controversial and poorly understood. The objective of this study was to assess the impact of whole-body CT compared with selective CT on mortality and management of patients with severe blunt trauma.

**Methods:**

The FIRST (French Intensive care Recorded in Severe Trauma) study is a multicenter cohort study on consecutive patients with severe blunt trauma requiring admission to intensive care units from university hospital trauma centers within the first 72 hours. Initial data were combined to construct a propensity score to receive whole-body CT and selective CT used in multivariable logistic regression models, and to calculate the probability of survival according to the Trauma and Injury Severity Score (TRISS) for 1,950 patients. The main endpoint was 30-day mortality.

**Results:**

In total, 1,696 patients out of 1,950 (87%) were given whole-body CT. The crude 30-day mortality rates were 16% among whole-body CT patients and 22% among selective CT patients (p = 0.02). A significant reduction in the mortality risk was observed among whole-body CT patients whatever the adjustment method (OR = 0.58, 95% CI: 0.34-0.99 after adjustment for baseline characteristics and post-CT treatment). Compared to the TRISS predicted survival, survival significantly improved for whole-body CT patients but not for selective CT patients. The pattern of early surgical and medical procedures significantly differed between the two groups.

**Conclusions:**

Diagnostic whole-body CT was associated with a significant reduction in 30-day mortality among patients with severe blunt trauma. Its use may be a global indicator of better management.

## Introduction

The availability of high-performance diagnostic imaging methods is a key element in the early diagnostic work-up of patients with severe blunt trauma. In the last two decades, the introduction of whole-body computed tomography (CT) has largely modified clinical practice in the management of patients with severe trauma and may influence surgical decisions. Recent technological advances related to the introduction of multislice CT led to increasing use of whole-body CT thanks to the reduction in data acquisition time and improvement in the quality of imaging data. However, the importance of this technology in early trauma management remains controversial. Besides its cost and the risk of radiation exposure, whole-body CT raises safety concerns about time delays due to patient transportation from the emergency room to the CT room and scanning [1-3].

To our knowledge, few studies have examined the benefit of whole-body CT on mortality in patients with major trauma and these yielded conflicting results [4-6]. One of these studies, performed by using the German Trauma Registry, suggested that whole-body CT may be associated with a reduction in severe trauma mortality [5]. However, the study's methodology, based on Trauma and Injury Severity Score (TRISS) and revised injury severity classification (RISC) approaches, is questionable because the calculation of both scores includes the Injury Severity Score (ISS) [7]. This finding may be due simply to a better detection of trauma lesions by whole-body CT, which increases the ISS and, consequently, the predicted mortality in this group. Furthermore, the lack of detailed information about in-hospital medical and surgical management did not allow the determinants of mortality reduction to be identified.

The FIRST (French Intensive care Recorded in Severe Trauma) study is a French observational prospective study that aimed at studying the impact of emergency care on hospital mortality of patients with severe blunt trauma. The collection of information about pre-hospital and hospital care, including diagnostic work-up, gave us the opportunity to examine the impact of whole-body CT compared with selective CT on blunt trauma mortality and to compare the global hospital management of the two studied groups.

## Materials and methods

### Study design

This analysis is an ancillary study of the FIRST epidemiological study which was conceived in order to prospectively gather pre-hospital and hospital data about patients with severe blunt trauma [8]. According to French law (law 88-1138, pertaining to biomedical research, 20 December 1988, modified on 9 August 2004 [9]), this non-interventional study did not require approval by an ethics committee or written informed consent from patients. The study was presented to and approved by the National Commission for Data Processing and Civil Liberties (authorization number 05-1059, confirmed on 24 February 2005). However, in accordance with French law, the intensive care unit (ICU) physician informed all patients or their families about the study. It involved ICUs and emergency departments from 14 university hospitals located throughout France (three centers in Paris, two in Lyon, and one each in Marseille, Nantes, Lille, Grenoble, Besançon, Nimes, Poitiers, Limoges, and Dijon).

As previously described, consecutive patients were recruited between December 2004 and March 2007 if they were at least 18 years old and had a severe blunt trauma defined as trauma requiring admission into an ICU within 72 hours after injury or, in the case of early death before ICU admission, trauma managed by a mobile ICU (MICU). Exclusion criteria were (a) penetrating traumas and (b) deaths occurring before the implementation of any advanced life-sustaining treatment. A total of 3,205 patients were eligible for inclusion in the FIRST study. Patients with incomplete or poor-quality data regarding hospital of first admission, ISS, pre-hospital management, or vital status were secondarily excluded (*n *= 502), leading to a FIRST study sample of 2,703 patients.

**Figure 1:**
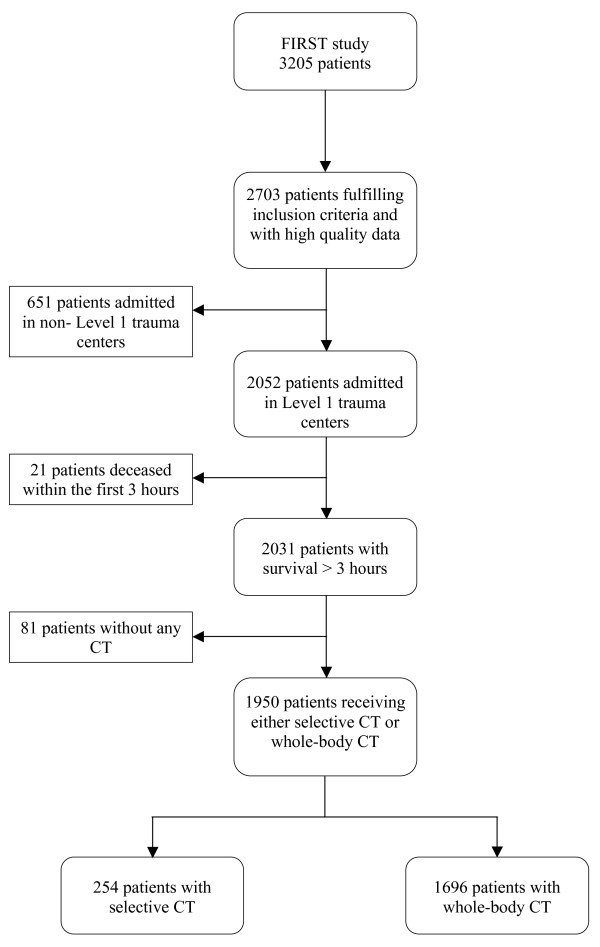
**Study flow chart**. CT, computed tomography; FIRST, French Intensive care Recorded in Severe Trauma.

For the purpose of the present analysis, further exclusion criteria were retained (Figure 1). First, 651 patients initially admitted in non-university hospitals before their admission in a university hospital ICU were discarded because information about CT could not be reliably collected in these patients. To guarantee that all patients had had a chance to undergo either whole-body or selective CT of one or more body regions, 21 patients who died within the first three hours after the accident were excluded (five patients with whole-body CT, three patients with selective CT, and 13 patients without any CT). Furthermore, 81 patients who did not receive any CT were excluded in order to limit indication bias. Thus, 1,950 patients were retained in the present observational analysis and were divided into two groups: those who benefited from a whole-body CT and those who benefited from a selective CT according to the diagnosis strategy defined by each trauma team.

### Data collection

ICU physicians collected data from the medical records of MICUs, emergency departments, and ICUs. In each center, ICU physicians aided by local research assistants entered data into the FIRST database that is hosted by the Clinical Investigation Center in Dijon. The Clinical Investigation Center was responsible for logistic coordination of the study, data quality control, and statistical analysis.

ICU physicians collected (a) patients' characteristics; (b) data about accident circumstances; (c) hospital units involved in the early care of patients before admission to the ICU; (d) clinical and biological data on the pre-hospital phase if available, upon hospital admission, and 24 and 72 hours after the trauma; and (e) a summary of clinical variables at patient discharge or death.

During the pre-hospital phase, the following data were recorded: initial physiological variables (arterial pressure, respiratory rate, and oxygen saturation as measured by pulse oximetry), pupil status, Glasgow Coma Scale [GCS] score, and life-sustaining treatments (venous line, fluid loading and catecholamine administration, tracheal intubation, ventilation, blood products, and chest tube).

Information on physiological variables and life-sustaining treatments was also collected upon arrival at the first hospital and 24 and 72 hours after the accident. The first available measurement, either at the pre-hospital phase or upon hospital admission, was used to describe the initial physiological status of the patient. At patient discharge from the ICU or death (within 30 days), anatomic injury diagnoses with corresponding Abbreviated Injury Scale (AIS) codes and the ISS were recorded from medical records. The AIS was coded according to the 1998 updated classification [10] by local research assistants using medical, radiological, and surgical reports. Local ICU physicians reviewed all problematic cases.

Several variables regarding the accident circumstances or the initial medical trauma assessment of the patient were important to take into consideration because they could influence imaging strategy. Two variables dealing with either the severity of accident or the suspected severity of trauma were constructed [11,12]. The accident was considered potentially severe if, in the case of a road traffic accident, at least one of the following points was recorded: pedestrian, no safety equipment (air bag, seat belt, crash helmet, and so on), excessive speed, victim ejected/crushed/burned/cut free from the vehicle, death of other victims in the vehicle, and vehicle fall of more than 6 meters. For the other accidents, severity was defined as a fall of more than 6 meters or crushing by lifting or agricultural equipment. Trauma was defined as potentially serious if, at the initial medical examination, there was suspicion of fractured skull, fractured pelvis, flail chest, or spinal injury or the presence of limb amputation, severe burns, or smoke inhalation. Because pre-hospital and hospital management may depend on the accident time during on-call periods, we defined two variables related to accident time: daytime (from 8:30 a.m. until 6:30 p.m.) versus night-time and weekend (from Saturday 1 p.m. until Monday 8 a.m.) versus other days.

All surgical procedures received by patients until ICU discharge were recorded and coded by physicians at the coordination center. Hemostatic procedures included arterial embolization and hemostatic thoracotomy or abdominal laparotomy. Orthopedic procedures included all types of bone fixation of upper and lower limbs.

Whole-body CT was an unenhanced CT of the head followed by contrast-enhanced CT of the chest, abdomen, pelvis, and complete spine. Information about whole-body and selective CT to one or more of these body regions was recorded in the first clinical department where the patient was admitted (emergency, surgical, or radiology unit or ICU) and, if needed, in subsequent departments that received the patient until his or her admission to an ICU. All other imaging procedures were recorded in a similar way. The main outcome measurement was the vital status at 30 days or at ICU discharge if discharge occurred within the first 30 days.

### Statistical methods

Comparisons of patients given a whole-body CT with those given a selective CT were performed by using chi-squared tests or, if needed, Fisher exact tests. To address selection and confounding biases that could not be totally controlled by the exclusion of patients who did not have any kind of CT and to assess the mortality reduction risk by using the initial whole-body CT, we constructed a propensity score. This approach is based on the idea that the probability of undergoing either whole-body or selective CT may depend on the patient's age, sex, study center, accident circumstances, initial medical assessment, and physiological status as well as on the administration of life-sustaining treatments during the pre-hospital phase or at hospital admission. We computed a non-parsimonious logistic regression model that included 24 potentially relevant covariates regarding the use of either whole-body or selective CT (variables listed in Table 1) [13]. The predicted probability that was derived from the logistic equation defined the propensity score for each patient. The discriminative power of the propensity score was quantified by the c statistic corresponding to the area under the receiver operating characteristic (ROC) curve. The quality of the propensity score was confirmed by checking the balance of covariates among patients with whole-body CT and among those with selective CT after adjustment for the propensity score.

**Table 1:** Baseline characteristics of patients according to the extent of computed tomography

	Selective CT (*n *= 254)		Whole-body CT (*n *= 1,696)		*P *value	*P *value adjusted for propensity score
	Number	Percentage	Number	Percentage		
Age					< 0.001	0.59
< 25 years	56	22.0%	450	26.5%		
25 to less than 50 years	99	39.0%	797	47.0%		
≥ 50 years	99	39.0%	449	26.5%		
Sex					0.33	0.95
Women	68	26.8%	406	23.9%		
Men	186	73.2%	1,290	76.1%		
Initial systolic blood pressure					0.06	0.91
< 90 mm Hg	24	9.8%	226	13.4%		
90 to less than 110 mm Hg	35	14.3%	306	18.2%		
≥ 110 mm Hg	186	75.9%	1,151	68.4%		
Accident severity					< 0.001	0.07
Not severe	152	61.3%	487	29.5%		
Severe	96	38.7%	1,162	70.5%		
Road traffic accident					< 0.001	0.16
No	145	57.1%	560	33.0%		
Yes	109	42.9%	1,136	67.0%		
Hospital admission delay					0.04	0.90
< 1 hour	37	14.6%	198	11.7%		
1 to less than 3 hours	171	67.3%	1,267	74.7%		
≥ 3 hours	46	18.1%	231	13.6%		
Pre-hospital management					< 0.001	0.78
Non-MICU	37	14.6%	32	1.9%		
MICU	217	85.4%	1,664	98.1%		
Initial heart rate					< 0.001	0.39
≤ 50 beats per minute	20	7.9%	96	5.7%		
50 to less than 120 beats per minute	223	87.8%	1,333	78.6%		
≥ 120 beats per minute	11	4.3%	267	15.7%		
Pre-hospital fluid loading					< 0.001	0.56
No	97	39.4%	265	15.9%		
Yes	149	60.6%	1,401	84.1%		
Pre-hospital intubation					< 0.001	0.97
No	156	61.7%	779	46.2%		
Yes	97	38.3%	906	53.8%		
Pre-hospital catecholamine administration					0.07	0.81
No	230	91.6%	1,474	87.7%		
Yes	21	8.4%	207	12.3%		
Study center					< 0.001	0.81
Paris	12	4.7%	449	26.5%		
Besançon	14	5.5%	95	5.6%		
Dijon	23	9.1%	140	8.3%		
Grenoble	39	15.4%	271	16.0%		
Lille	26	10.2%	254	15.0%		
Lyon	26	10.2%	78	4.6%		
Nantes	13	5.1%	70	4.1%		
Nîmes	48	18.9%	29	1.7%		
Poitiers	15	5.9%	126	7.4%		
Limoges	15	5.9%	59	3.5%		
Marseille	23	9.1%	125	7.4%		

All variables listed in this table were included in the propensity score along with other variables not associated with whole-body computed tomography (CT) or selective CT: Glasgow Coma Scale score, suspected trauma severity, blood products, ventilation, day/night, accident time, weekend/other days, pre-hospital cardiac arrest, hemoglobin, and prothrombin ratio. MICU, mobile intensive care unit.

The impact of whole-body CT on mortality was assessed by using several multivariable logistic models. First, we used a classic model in which the CT variable and all covariates (baseline characteristics and post-CT characteristics related to medical treatment in the first 24 hours) associated with mortality at a significance level of less than 0.20 in bivariate analysis were introduced and selected through a backward procedure as described by Hosmer and Lemeshow [14]. Second, the propensity score was used in two different ways, either for regression adjustment or for matching [15,16]. The propensity score (either as a continuous variable or categorized according to quintiles) replaced baseline characteristics in the logistic regression. The same set of variables that related to post-CT treatments and that was used in the first multivariate logistic model was used as covariates. We also used propensity-based matching to produce adjusted estimates of the effect of whole-body CT on mortality. We performed a five-digit case control match on propensity score by using SAS™ version 9.1 (SAS Institute Inc., Cary, NC, USA). Each patient who had a whole-body CT was matched to one sole patient who had only a selective CT on five digits, then on four digits, and (if needed) on three, two, and one digit of the propensity score (the matching became rougher and rougher). The quality of matching was assessed by comparing baseline characteristics between both CT groups by using the chi-squared test or, if needed, the Fisher exact test. A logistic regression model adjusted for covariates related to post-CT treatments was also used for assessing the impact of whole-body scan on mortality. The goodness-of-fit of the various logistic regression models was assessed according to Akaike information criteria and the Hosmer-Lemeshow test.

To compare our results with those obtained in a previous study [5], we also used a TRISS-adjusted approach. The TRISS method is used to predict the probability of survival at discharge [17]. There were large differences in severity mix between our observed data set and the US prediction data set as reflected by the M statistic (M = 0.42 in the whole-body CT group and 0.51 in the selective CT group). Thus, to compare the observed survival with the TRISS-predicted survival, we calculated only the Ws score and corresponding Zs statistic [18]. These scores are produced by a method of direct standardization of the difference between the observed number of survivors and the TRISS-predicted number of survivors according to the case mix of injury severity of the US prediction database. A positive value of Ws associated with a Zs of greater than 1.96 indicates a significantly better survival than that defined by the prediction database.

Data were expressed as mean with standard deviation, median with interquartile (25th to 75th) range, or percentage. We performed the statistical analyses by using SAS™ version 9.1 (SAS Institute Inc.) and STATA version 11 (StataCorp LP, College Station, TX, USA) software. A *P *value of less than 0.05 was considered significant, and all *P *values were two-tailed.

## Results

### Baseline characteristics of patients according to use of whole-body or selective CT

Among the 1,950 patients who had severe blunt trauma and who had a CT examination, 1,696 (87%) had a whole-body CT and 254 (13%) had a selective CT. Among patients with selective CT, the body regions were head in 202 patients (80%), abdomen/pelvis in 105 (41%), cervical spine in 89 (35%), and thorax in 59 (23%). The proportions of patients who received Focussed Assessment Sonography for Trauma (FAST) imaging (abdominal ultrasonography and chest radiography) were not significantly different in patients with selective CT and in those with whole-body CT (19.2% versus 23.4%, respectively; *P *= 0.15). There were significant differences in baseline characteristics between the two groups (Table 1). Patients with whole-body CT were significantly younger and were victims of more potentially severe accidents and more specifically of road traffic accidents. These patients were more often managed by physicians from MICUs in the pre-hospital phase and were more rapidly admitted to university hospital trauma centers. Although the initial physiological status (except for heart rate) and GCS score were similar in the two groups, patients with whole-body CT had benefited from a more aggressive management (fluid loading, intubation, and catecholamine administration) in the pre-hospital phase or at admission. The use of whole-body versus selective CT also significantly depended on the study center (Table 1).

The propensity score constructed from 24 baseline characteristics fitted well with data as indicated by the good area under the ROC curve (c index of 0.83). Furthermore, all baseline characteristics that were significantly related to the use of whole-body versus selective CT in the univariate analysis were no longer significant after adjustment for the propensity score (Table 1).

### Impact of whole-body versus selective CT on mortality

**Figure 2:**
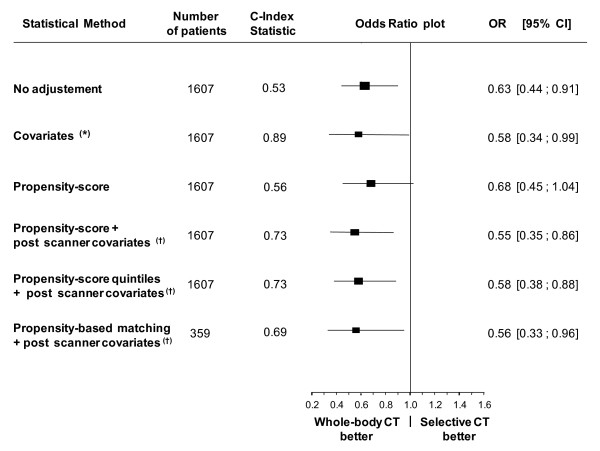
**Odds ratio for 30-day mortality associated with whole-body computed tomography (CT) by several adjustment methods**. C index statistic corresponding to the area under the receiver operating characteristic (ROC) curve *Multivariate logistic regression adjusted for pre-CT (age, hemoglobin, prothrombin ratio, ventilation, Glasgow Coma Scale, fluid loading, center, and pre-hospital cardiac arrest) and post-CT confounders (number of packed red blood cells in the first 24 hours and catecholamine administration in the first 24 hours). ^†^Multivariate logistic regression adjusted for post-CT confounders (number of packed red blood cells in the first 24 hours and catecholamine administration in the first 24 hours). CI, confidence interval; OR, odds ratio.

At day 30, 277 patients (16%) in the whole-body CT group and 56 (22%) in the selective CT group were deceased (absolute decrease of 6%, 95% confidence interval (CI) of 1% to 11%; *P *= 0.02). There was no significant difference between the two groups in the mortality rate at 24 hours (6.0% in the former group versus 8.3% in the latter). The adjusted impact of whole-body CT on 30-day mortality according to the various adjustment methods is summarized in Figure 2. Among the 1,607 patients for whom all variables were available, all methods (adjustment for pre-CT + post-CT covariates, propensity score + post-CT covariates, and propensity-based matching + post-CT covariates) led to a significant reduction of 30-day mortality in the whole-body CT group (Figure 2). The best model was the classic logistic regression model based on adjustment on pre- and post-CT covariates, as indicated by the area under the ROC curve (c statistics = 0.89) and the Hosmer-Lemeshow test (*P *= 0.54).

Cerebral death was the main cause of death and was significantly less frequent in the whole-body CT group than in the selective CT group (62% versus 79%, *P *= 0.016). The other main causes of death - hemorrhagic shock (14% versus 13%) and multivisceral organ failure (8% versus 2%) - were similar in the two groups.

### Impact of whole-body CT on mortality using the TRISS-adjusted method

The TRISS method was applied to 1,864 patients with an available revised trauma score. The TRISS-predicted mortality rates were 30% for the 1,635 patients who received whole-body CT and 22.7% for the 229 patients who received only selective CT. After standardization according to the case mix of injury severity of the US prediction database, the Ws and Zs scores were, respectively, 3.3 (95% CI 1.9 to 4.6) and 4.81 (*P *< 0.0001) among whole-body CT patients, indicating a significantly better survival in these patients than that predicted by the TRISS method. Corresponding Ws and Zs scores among selective CT patients were, respectively, 0.3 (95% CI -2.9 to 3.5) and 0.17 (*P *= 0.44), indicating the lack of significant difference between the observed and the TRISS-predicted survival in the latter group.

### Injury assessment among whole-body CT patients and selective CT patients

Compared with patients with selective CT, the proportion of whole-body CT patients with an AIS score of at least 4 was significantly higher for the thorax (*P *< 0.001) and spinal areas (*P *= 0.05) as well as for the lower limbs (*P *< 0.001) (Table 2). Similarly, the proportion of patients with an ISS of at least 35 was significantly higher for patients given whole-body CT (25.8%) than for those given selective CT (7.5%). The introduction of ISS as covariate in regression models shown in Figure 2 led to an increase in the mortality benefit associated with the use of whole-body CT. After adjustment for ISS, the odds ratio (95% CI) decreased from 0.58 (0.34 to 0.99) to 0.45 (0.26 to 0.77) in the classic logistic regression model and from 0.55 (0.35 to 0.86) to 0.42 (0.27 to 0.67) in the propensity logistic regression model.

**Table 2:** Abbreviated Injury Scale and Injury Severity Score among patients with whole-body or selective computed tomography

	Selective CT (*n *= 254)		Whole-body CT (*n *= 1,696)		*P *value
	Number	Percentage	Number	Percentage	
AIS					
Head					0.07
< 4	134	52.8	995	58.7	
≥ 4	120	47.2	701	41.3	
Neck					0.62
< 4	254	100	1,687	99.5	
≥ 4	0	0	9	0.5	
Abdomen					0.72
< 4	238	93.7	1,579	93.1	
≥ 4	16	6.3	117	6.9	
Thorax					< 0.001
< 4	216	85.0	1,175	69.3	
≥ 4	38	15.0	521	30.7	
Spine					0.05
< 4	240	94.5	1,540	90.8	
≥ 4	14	5.5	253	9.2	
Lower limb					< 0.001
< 4	251	98.8	1,594	94.0	
≥ 4	3	1.2	102	6.0	
Upper limb					1
< 3	254	100	1,695	99.9	
≥ 3	0	0	1	0.1	
Face					0.20
< 4	251	98.8	1,687	99.5	
≥ 4	3	1.2	9	0.5	
ISS					< 0.001
< 25	130	51.2	642	37.9	
25-34	105	41.3	617	36.4	
≥ 35	19	7.5	437	25.8	

AIS, Abbreviated Injury Scale; CT, computed tomography; ISS, Injury Severity Score.

### Comparison of therapeutic procedures until discharge among whole-body CT patients and selective CT patients

As shown in Table 3, the proportion of patients undergoing surgery was significantly higher among the whole-body CT group than in the selective CT group both within the first 24 hours (67% versus 59%, *P *< 0.02) and until ICU discharge (73% versus 64%, *P *< 0.001). The main differences between groups in surgical procedures used within the first 24 hours were the higher percentage of patients with hemostatic or orthopedic surgery in the whole-body CT group than in the selective CT group (*P *= 0.03 and *P *< 0.001, respectively). In contrast, more patients had undergone intracranial surgery at 24 hours in the selective CT group than in the whole-body CT group (*P *< 0.001). Furthermore, early medical management, as reflected by the use of intubation, blood transfusion, and catecholamines, was significantly more aggressive in patients given whole-body CT. A similar pattern was observed in surgical procedures until discharge, with significantly more hemostatic (*P *= 0.02) and orthopedic (*P *< 0.001) surgery and less intracranial surgery (*P *< 0.001) for patients given whole-body CT than in those given selective CT. Furthermore, spinal surgery (*P *= 0.01) and the use of a thoracic drain (*P *< 0.001) were significantly more frequent in the whole-body CT group.

**Table 3:** Surgical procedures among patients with whole-body or selective computed tomography

	Selective CT (*n *= 254)		Whole-body CT (*n *= 1,696)		*P *value
	Number	Percentage	Number	Percentage	
Within the first 24 hours					
All surgical procedures	152	59.8	1,142	67.3	0.02
Hemostatic surgery	16	6.3	187	11.0	0.03
Abdominal surgery	15	5.9	136	8.0	0.24
Thoracic surgery	4	1.6	29	1.7	1
Intracranial surgery	35	13.7	68	4.0	< 0.001
Spinal surgery	12	4.7	117	6.9	0.20
Orthopedic surgery	42	16.5	519	30.6	< 0.001
Medical procedures					
Intubation	148	58.5	1,232	72.6	< 0.001
Packed red blood cells ≥ 4	36	14.2	416	24.5	< 0.001
Platelets	22	9.3	221	14.2	0.04
Catecholamines	95	38.5	888	53.4	< 0.001
Until discharge					
All surgical procedures	163	64.2	1,238	73.0	< 0.001
Hemostatic surgery	20	7.9	227	13.4	0.02
Abdominal surgery	22	8.7	188	11.1	0.25
Thoracic surgery	4	1.6	39	2.3	0.47
Thoracic drain	20	8.3	307	18.3	< 0.001
Intracranial surgery	37	14.6	95	5.6	< 0.001
Spinal surgery	12	4.7	164	9.7	0.01
Orthopedic surgery	47	18.5	594	35.2	< 0.001

CT, computed tomography.

## Discussion

The main finding of this observational study was the significant reduction in 30-day mortality among patients who had a whole-body CT for early assessment of blunt trauma in comparison with patients who had only selective CT. According to the adjustment method, the relative risk reduction ranged from 0.42 to 0.45.

The main strength of this prospective study was to deal carefully with potential indication biases of whole-body CT. First, we excluded patients who did not survive long enough to undergo a whole-body CT. This precaution was taken because whole-body CT may be more time-consuming than selective CT or other diagnostic methods used in the emergency room (or both) and thus may not be proposed to the most severely injured patients [19,20]. Second, we also excluded patients who did not receive any CT. Compared with patients with whole-body or selective CT, these patients had a high probability of presenting specific initial characteristics likely to influence the outcome. Indeed, patients without any CT showed a higher GCS score but more unstable hemodynamic status, leading to a high rate of mortality within 24 hours (25%) or until discharge (37%). Third, although the initial physiological status was relatively similar between whole-body CT patients and selective CT patients, the two groups presented significant differences in regard to some characteristics related to the accident or pre-hospital management. Compared with patients with selective CT, patients with whole-body CT were younger, had had a more serious accident, and had received a more intensive treatment in the pre-hospital phase. In contrast, there were no significant differences between the two groups in the GCS score, the suspected severity of trauma, the accident time, or the use of blood products in the pre-hospital phase or at admission. Fourth, to limit a possible indication bias, we used several adjustment methods, including the construction of a non-parsimonious propensity score, for controlling not only *a priori *characteristics that may influence both the probability of receiving whole-body versus selective CT and the risk of death but also *a posteriori *variables (regarding medical treatment in the first 24 hours) related to the risk of death. Interestingly, we obtained very consistent results using either a classic multivariate logistic regression model (the best model according to c statistics and the Hosmer-Lemeshow test) or logistic regression models based on propensity score considered as a continuous variable, a variable categorized according to score quintiles or a matching variable.

Our study supports and extends the results of the German Trauma Registry-based study [5]. Using a TRISS-based methodology, the German study concluded that the integration of whole-body CT in early trauma care significantly increased the probability of survival in patients with severe trauma. However, in contrast to our study, this study did not show any significant difference in crude mortality rates between patients who received whole-body CT and those who did not. Furthermore, the results of this observational study were adjusted only for hospital level, year of trauma, and center and thus did not take into account the main severity bias associated with the indication of a CT, so that no causal inference can be made. The TRISS method is commonly used to assess the management of severe blunt trauma [21-23]. In the FIRST study, we also observed a better survival of whole-body CT patients than that predicted by TRISS, whereas there was no difference in survival of selective CT patients. However, the use of the TRISS method is questionable for evaluating the impact of whole-body CT on mortality. Indeed, the TRISS equation is based on the ISS, which depends on whether or not patients were given whole-body CT. As noted in a previous review on the topic, poorer ISS due to improvement in lesion detection by whole-body CT will lead to an increased predicted mortality and thus to erroneous conclusions regarding the benefit of whole-body CT [7]. Another study showed that ISS failed to differentiate severe injury from mismanagement of injury. Because the ISS mixes outcome data with injury severity, it incorrectly assigns increased severity to the lesser injuries of mismanaged patients [24].

The interpretation of the association between whole-body CT and ISS is uncertain. Clearly, patients given whole-body CT had higher ISS than patients given selective CT. The first explanation may be that patients presumed to have more severe injuries at admission were more likely to receive whole-body CT. If this was the case, ISS should have been included either in the propensity score or in adjustment variables. However, we did not find any association between the use of whole-body CT and the suspected severity of trauma. The second explanation is that whole-body CT led to a better detection of lesions than selective CT and thus to higher ISS. In that case, adjustment for ISS, which is strongly related to the risk of death, may result in an overestimation of the beneficial impact of whole-body CT. This reason led us to judge the TRISS method inappropriate in the study context and to decide to exclude ISS from the propensity score and adjustment variables. Nevertheless, using a conservative strategy based solely on *a priori *factors likely to influence the choice of the imaging methods, we were able to highlight a pronounced reduction in 30-day mortality for patients given whole-body CT.

The reasons why the use of whole-body CT may induce a reduction in 30-day mortality in patients with severe trauma are difficult to unravel in an observational study. Our study revealed more intensive pre-hospital management as reflected by more frequent on-scene intubation and higher fluid loading and continuous intravenous catecholamine infusion in the whole-body CT group in comparison with the selective CT group. Early management of hypoxemia and hypotension can reduce the risk of early fibrinolysis and prevent patients from being admitted with clinical coagulopathy [25,26]. On the other hand, in our observational cohort, the main components of pre-hospital treatment were differentially associated with the risk of death. On-scene intubation and continuous intravenous catecholamine infusion, which probably reflect the higher severity of trauma, increased the risk of death whereas fluid loading decreased the risk. Theoretically, differences in early medical pre-hospital management have been taken into account in our adjustment strategy. However, we cannot exclude residual confounding regarding pre-hospital medical management, which may explain the risk reduction in 30-day mortality.

We did not find any significant difference between groups in the early mortality rate, suggesting no major differences in their initial clinical status but rather a later deterioration of patients from the selective CT group. This may be due to unrecognized injuries or delayed in-hospital management or both. In our study, hospital surgical strategies were available within the first 24 hours and at discharge. During the first 24 hours, whole-body CT patients benefited from more frequent surgical management and more intensive life-sustaining treatments characterized by more frequent transfusion, intubation, and catecholamine infusion. After a whole-body CT, this overall dynamic therapeutic approach may reduce preventable deaths. Indeed, many deaths are due mainly to incomplete or poor assessment of organ injuries, delayed decision of surgical operation, delayed hemostasis intervention, or errors in resuscitation procedures [27-30]. Overall, hemostatic surgery was more frequent in the whole-body CT group than in the selective CT group. The lack of difference in regard to abdominal and thoracic surgery suggests that the quality of bleeding detection and radiological hemostasis played a major role in outcome benefit for whole-body CT patients [31-33]. Severe thoracic injury may increase perioperative instability and thus the risk of perioperative events. Although whole-body CT patients presented more severe thoracic lesions, they benefited more frequently from early orthopedic surgery. This suggests that improved management of thoracic injuries, including more frequent chest tube insertion, could help the trauma team to accelerate access to surgical treatment [34].

Another explanation lies in the higher proportion of cerebral death in the selective CT group than in the whole-body CT group. Eighty percent of selective CT patients underwent head CT. These patients tended to have more severe cerebral lesions (AIS score of at least 4) and had significantly more frequent early neurosurgical intervention. Head injury is known to be the single largest contributor to trauma center deaths [35]. Other studies have shown that extensive intracranial bleeding requiring neurosurgical intervention is associated with a substantially higher probability of in-hospital mortality in comparison with non-surgical intracranial bleeding [36,37]. Furthermore, whole-body CT patients were significantly younger than selective CT patients. Although our analyses were adjusted for age, we were unable to control other age-related factors such as pre-existing platelet anti-aggregant or anti-coagulant treatments (or both) that predispose patients to bleeding, especially in brain injury, and have a negative impact on survival [8,38,39]. This raises the hypothesis that the worse outcome of selective CT patients may be due to an effect of the cerebral injury and not to lesion misdetection. Patients with extremely severe injury are already known to have a low probability of surviving [40].

Our study also presents several limitations. This was an observational cohort, so that, despite our careful adjustment strategy, we cannot rule out residual confounding effects and thus a causality link cannot be definitely demonstrated. In particular, we have no information about scanning protocols or type of scanners used, so that possible variations in CT protocols between centers cannot be excluded. We have no reason to suspect major between-center differences in whole-body CT indications, since, in France, whole-body CT is systematically recommended unless severe trauma patients present an unstable hemodynamic status or severe isolated head injury or both. In addition, in all trauma centers, scans are first interpreted by radiologists and further reviewed by clinicians in charge of the patients (emergency physicians or surgeons or both). Because the FIRST study was not specifically designed to address this topic, we have no details about the time elapsed between admission in a university hospital trauma center and diagnostic imaging work-up. However, since more than 80% of the patients received whole-body or selective CT in the first unit of admission (emergency room, surgical unit, or ICU), we can hypothesize that all patients were examined within the first 24 hours and that the majority of them were examined in the first two hours after their admission. Furthermore, because the quality of data regarding diagnostic imaging was uncertain for patients initially admitted in general hospitals before their transfer to university hospital trauma centers, these patients were excluded from the present analysis. Thus, our results can be extrapolated only to severe trauma patients admitted directly to university hospital trauma centers. Lastly, the design of the FIRST study did not take into account patients with penetrating trauma or pediatric trauma patients.

## Conclusions

Our prospective study showed that initial whole-body CT was associated with a significant 30-day mortality reduction that could be related to higher detection of traumatic lesions and higher use of surgical treatment. However, our study stressed the important contribution of severe head injury for explaining the lower mortality in patients who received selective CT. Alternatively, whole-body CT may be only an overall indicator of better pre-hospital and hospital management of patients with severe blunt trauma. Clearly, only a randomized controlled trial could solve the issue but its feasibility is highly questionable in the present state of diagnostic practices in severe trauma.

## Key messages

• Initial whole-body computed tomography (CT) is used for a large majority of patients with severe blunt trauma.

• Whole-body CT is associated with a significant reduction in 30-day mortality. This reduction is due mainly to a lower proportion of cerebral death.

• The beneficial impact of whole-body CT on mortality is independent of the initial physiological status.

• Surgical management was more frequent among patients with whole-body CT. Whether this could explain the reduction in mortality remains unclear.

## Abbreviations

AIS: Abbreviated Injury Scale; CI: confidence interval; CT: computed tomography; FIRST: French Intensive care Recorded in Severe Trauma; GCS: Glasgow Coma Scale; ICU: intensive care unit; ISS: Injury Severity Score; MICU: mobile intensive care unit; ROC: receiver operating characteristic; TRISS: Trauma and Injury Severity Score.

## Competing interests

The authors declare that they have no competing interests.

## Authors' contributions

J-MY and AY conceived of this study with considerable help from CB-K and MF for sequence alignment. BR gave support for proofreading of this paper, was involved in the initiation and design of the study, participated in the acquisition of data, and contributed to the interpretation of data and final revision of the manuscript. J-MY was involved in the study design and in the acquisition, analysis, and interpretation of data and wrote the first draft of the manuscript. AY participated in the conception of this analysis, the interpretation of data, and draft writing. DG, CJ, and CM participated in the design of the study, the acquisition of data, and the final revision of the manuscript. CB participated in the design of the study and performed the statistical analysis. CB-K was responsible for the logistic coordination of the study; was involved in the design of the study, statistical analysis; and interpretation of data, and helped to draft the manuscript. MF initiated and coordinated the FIRST study and was involved at all steps of the study. All authors read and approved the final manuscript.
